# Irradiation-Assisted Enhancement of Foaming and Thermal Gelation Functionality of Liquid Egg White

**DOI:** 10.3390/foods13091342

**Published:** 2024-04-26

**Authors:** Yan Zhang, Jianying Zhao, Lichao He, Jin Zhu, Yue Zhu, Guofeng Jin, Ruihang Cai, Xiaola Li, Chengliang Li

**Affiliations:** 1 Key Laboratory of Geriatric Nutrition and Health (Ministry of Education), China Food Flavor and Nutrition Health Innovation Center, School of Food and Health, Beijing Technology and Business University, Beijing 100048, China; 2 Department of Tea and Food Science and Technology, Jiangsu Vocational College of Agriculture and Forestry, Jurong 212400, China; 3 Zhejiang Institute of Subtropical Crops, Zhejiang Academy of Agricultural Sciences, Wenzhou 325005, China; 4 College of Food Science & Technology, Huazhong Agricultural University, Wuhan 430070, China

**Keywords:** irradiation, egg white protein, protein fragmentation, protein oxidative aggregation, structural characteristics, foam and gels

## Abstract

Ionizing radiation has its unique popularity as a non-thermal decontamination technique treating with protein-rich foodstuffs to ensure the microbial and sensory quality, particularly for shell eggs. However, the changes in the functional properties of egg protein fractions such as liquid egg white (LEW) with macro/microstructural information are still controversial. Hence, this study was designed to elaborate the foaming and heat-set gelation functionality of LEW following different γ-ray irradiation dose treatments (0, 1, 3 or 5 kGy). For such, the physicochemical properties (active sulfhydryl and the hydrophobicity of protein moieties), structural characteristics (through X-ray diffraction, Fourier-transform infrared spectroscopy and differential scanning calorimetry) and interfacial activities (rheological viscosity, interfacial tension, microrheological performance) were investigated. Then, the thermal gelation of LEW in relation to the texture profile and microstructure (by means of a scanning electron microscope) was evaluated followed by the swelling potency analysis of LEW gel in enzyme-free simulated gastric juice. The results indicated that irradiation significantly increased the hydrophobicity of liquid egg white proteins (LEWPs) (*p* < 0.05) by exposing non-polar groups and the interfacial rearrangement from a β-sheet to linear and smaller crystal structure, leading to an enhanced foaming capacity. Microstructural analysis revealed that the higher dose irradiation (up to 5 kGy) could promote the proteins’ oxidation of LEW alongside protein aggregates formed in the amorphous region, which favored heat-set gelation. As evidenced in microrheology, ≤3 kGy irradiation provided an improved viscoelastic interface film of LEW during gelatinization. Particularly, the LEW gel treated with 1 kGy irradiation had evident swelling resistance during the times of acidification at pH 1.2. These results gave new insight into the irradiation-assisted enhancement of foaming and heat-set gelation properties of LEW.

## 1. Introduction

Liquid egg whites (LEWs) offer notable benefits in terms of their high nutritional value and superior bioavailability, making them a valuable resource for utilization in the food industry and sports nutrition [1]. The versatile nature of egg white lends itself well to a variety of convenience food applications, including bakery items and surimi products, owing to its exceptional functional attributes such as foaming and gelling properties [2]. Additionally, egg white proteins, consisting of ovalbumin (54%), lysozyme (3.4%), ovomucin (3.5%), ovotransferrin (12%) and ovomucoid (11%), and their derivative peptides have been fundamentally utilized as functional and nutritional ingredients in the food and biomedical industry [3,4]. LEW also has limited applications due to the short shelf-life and transportation issues. So, egg protein powders are extensively prepared during practical manufacturing via spray- and free-drying approaches [5]. Despite these facts, the inherent foaming and gelling properties of natural liquid egg white proteins (LEWPs) may not fully satisfy the growing demands of food processing and nutrient production. Consequently, there is a pressing need to enhance the functional properties of LEW in a manner that is both effective and health-conscious.

The structure, surface hydrophobicity, thermal properties and aggregation of proteins are important factors that affect their functionality. The foaming characteristics are primarily influenced by the capacity of proteins being absorbed to the gas–liquid interface through hydrophobic interactions during bubbling, as well as by their capability to form a viscoelastic layer following partial protein unfolding [3]. However, the foaming properties of LEWPs can be adversely affected by the heating process due to their heat-sensitive characteristics. Hence, a scale of physical applications have been extensively used to modify and improve the functional characteristics of LWP, such as supercritical carbon dioxide, ultrasound or high-pressure homogenization [6]. These non-thermal methods mainly work by increasing the size of dispersed particles and reducing the viscosity of egg white proteins. In addition, physical treatments can also impact the denaturation and structural modifications of egg white proteins, leading to changes in gel properties which usually involve the gradual aggregation of denatured proteins mainly driven by the modification of secondary structure and hydrophobic interactions [7,8]. These findings suggest that utilizing physical techniques to enhance the functionality of egg white shows promise, but further research is needed to investigate the inter-relationship between the protein structure, interface formation and functional changes during physical treatment approaches.

During the latest decade, ionizing radiation, particularly γ-ray irradiation, has been primarily employed as a non-thermal food sterilization technique for preservation purposes [9]. This technology generates free radicals (i.e., hydrated electron, hydrogen atom, hydroxyl radical, etc.) by breaking down water molecules through radiolysis, consequently impacting the dispersive characteristics of food proteins by protein fragmentation, denaturation/modification and inducing oxidative crosslinking or aggregation [10,11]. Consequently, irradiation has gained popularity for its ability to enhance various functional attributes of food proteins, e.g., soybean proteins, insect proteins and meat emulsion [12,13,14]. Studies have indicated that irradiation is highly effective to control foodborne pathogens (e.g., *Salmonella* and *Campylobacter*) in shell eggs and egg products [15,16]. The United States Food and Drug Administration (US FDA) has implemented and approved the use of irradiation up to 3 kGy to control *Salmonella* in fresh shell eggs since 2000 [17]. Previous studies have shown that ≥3 kGy γ-ray irradiation can enhance the emulsion creaming capacity and stability of LEWPs mainly due to the modification of the secondary structure and interface adsorption with a smaller droplet size [18]. Nevertheless, the underlying mechanisms of γ-ray irradiation treatment on the foaming and gelation properties of LEWPs remain inadequately understood. Therefore, the primary objective of this study was to examine the structural alteration of LEWPs and the impact of protein hydrodynamic properties on the evolution of foaming and heat-set gel properties following the treatment of varying doses of γ-ray irradiation (0, 1, 3, 5 kGy).

## 2. Materials and Methods

### 2.1. Egg Separation and Preparation of LEW

Fresh eggs were purchased from a local agricultural product market, and the eggshell surface was cleaned with tap water. The egg white and yolk were separated using an egg separator. The resulting LEW was stirred evenly with a magnetic blender and sealed with polyethylene bags for subsequent use.

### 2.2. Pasteurization of LEW

The packaged LEW samples were immediately sent to the Irradiation Center of Institute of Food Science and Technology CAAS (Beijing, China) for irradiation treatment. The packed LEW samples were carefully placed in insulated boxes containing ice and were irradiated with 1, 3 and 5 kGy, respectively, at a dose rate of 3.6 kGy/h; Amber Perspex 3042 dosimeters (Atomic Energy Research Establishment, Harwell, Oxfordshire, UK) were used to measure the radiation dose. After irradiation, the samples were immediately transported to the laboratory and placed in a refrigerator at 4 °C (storage for up to 3 d).

### 2.3. Preparation of LEW Emulsion and Gels

According to Liu et al. with slight modification [18], 10 g of the LEW sample was accurately mixed in 90 mL pure water and a small amount of sodium azide, and finally 30 mL soybean oil was added. Samples were then homogenized for 3 min at 8000 rpm using a homogenizer (XHF-DY, Scientz, Ningbo, China) at room temperature. The upper bubbles were filtered with 100-mesh gauze, and emulsion samples were put in a 4 °C freezer. Additionally, 20 mL of LEW samples was placed in a 25 mL plastic cup, sealed with cling film, then heated in a 90 °C water bath pot, then immediately cooled in ice water. Samples were finally put in a refrigerator at 4 °C for 12 h towards gelatinization for the follow-up characterization.

### 2.4. Surface Hydrophobicity and Free Sulfhydryl Determination

The surface hydrophobicity of LEWPs was determined by the 1-(anilino)naphthalene-8-sulfonate (ANS^−^) fluorescence probe method [19]. The LEW samples were homogenized at 2000 rpm for 1 min, and the resulting solution was diluted as different concentration gradients with 0.1 M phosphate buffer. A total of 10 mL of the sample solution for each group was taken and added with 50 μL of 8 mol/L ANS solution. The mixture was agitated using a vortex and stood for 10 min. Afterwards, the fluorescence intensity (FI) was measured using a fluorescence spectrophotometer with excitation wavelength 330 nm and emission wavelength 490 nm. The protein concentration of samples was determined by the Bradford method [20]. The surface hydrophobicity index (H_0_) was determined using the slope of extrinsic fluorescence vs. protein concentration. In addition, Ellman’s reagent (5,5′-dithiobis(2-nitrobenzoic acid, DTNB) was used to determine the –SH groups in different LEW samples [21]. Briefly, each 1.5 g of egg white was diluted with 10 mL 0.1 M Tris-glycine buffer (pH 8.0, containing 4 mM EDTA, 0.09 M glycine and 0.5 M sodium chloride), and then 0.1 mL resulting solution was vortex-mixed with 2.9 mL 0.5% SDS solution (containing 0.02 mL Ellman’s reagent). After reaction in dark for 30 min at room temperature, the absorbance of the assayed sample was measured at 412 nm by a spectrophotometer (UV-1800, MAPADA, Shanghai, China). A solution without Ellman’s reagent was used as a blank. The protein content in all samples was determined by the Bradford method, and the content of free –SH groups was calculated by the following equation:

μM SH/g protein = 73.53 × A_412_ × D/C

where 73.53 = 10^6^/(1.36 × 10^4^), 1.36 × 10^4^ (M^−1^ cm^−1^) is the molar extinction coefficient, A_412_ is the absorbance of the sample, D is the dilution ratio and C is the protein concentration.

### 2.5. Determination of Foaming Capacity and Stability

The foaming ability (FA) and foaming stability (FS) of irradiated LEW samples were determined according to the method of Sheng et al. [22]. Foams were produced of a 100 mL (V_0_) LEW sample using a laboratory homogenizer (IKA RW-20, Staufen, Germany) at 1500 rpm for 90 s in a 500 mL cylinder. The final volume of egg white foam after whipping and the liquid volume drained from the foam after 30 min were designated as V_1_ and V_2_, respectively. The FC and FS were calculated using the equations as follows:

FA (%) = 100 × V_1_/V_0_

FS (%) = 100 × V_2_/V_0_

### 2.6. Rheological Viscosity Determination

The rheological viscosity of LEW samples irradiated at different doses was measured using a rotational rheometer (DHR-2, Waters Corporation, Milford, MA, USA) by a steady shear test. Firstly, LEW samples were balanced at room temperature for 20 min. Then steady-state scanning was used with a conical plate (40 mm diameter, 0.052 mm gap). The experimental temperature was set at 25 °C, and the shear rate ranged from 1 s^−1^ to 700 s^−1^.

### 2.7. Interfacial Tension Determination

The dynamic surface tension of LEW samples was measured by a surface tensiometer (K100, Krüss GmbH, Hamburg, Germany) using the Wilhelmy’s plate method at room temperature for 40 min [23]. For each measurement, an aliquot volume of the LEW sample (30 mL) was taken in a 50 mL beaker, and 40 data points were recorded based on the droplet profile. The surface tension was thus calculated according to Laplace’s equation and plotted versus time used for analysis.

### 2.8. Temperature-Programmed Microrheological Measurement of LEW Emulsion

Based on the principle of diffusion wave spectroscopy (DWS), the rheological properties of different LEW emulsion samples (refer to Section 2.3) were measured by a microrheometer (Rheolaser Master™, Formulaction, Toulouse, France). The temperature during the assay ranged from 30 to 90 °C, the time was set at 0–1 h and the heating rate was 1 °C/min. The motion of the droplet within the sample was quantified as the mean square displacement (MSD) of the scatterer. The resulting parameters, i.e., the elasticity index (EI) and macroscopic viscosity index (MVI), were obtained through Rheosoft Master 1.4.0 software analysis.

### 2.9. Crystalline Analysis of LEW Gel

X-ray diffraction patterns of freeze-dried LEW gel powder were obtained using a diffractometer (D/MAX 2500 V, Rigaku Corporation, Tokyo, Japan) at 25 °C. The samples were laid flat in the circular hole on the test piece and scanned in the 2θ range 10–80° at a 10° min^−1^ scan rate, with Ni filtered Cu Kα radiation (λ = 0.1542 nm) at settings of 40 kV and 40 mA.

### 2.10. Protein Molecular Conformation Examination of LEW Gel

Briefly, freeze-dried LEW gel powder was carefully prepared and mixed with potassium bromide at a ratio of 1:50 (*w*/*w*) and was ground in an agate mortar, crushed into a transparent sheet and subjected to formal analysis in a Fourier-transform infrared (FT-IR) spectrometer (Nexus 470, Nicolet Corporation, Madison, WI, USA) with a scanning range of 4000–400 cm^−1^. Before determination, the sample was scanned with air and the background interference was deducted during calculation.

### 2.11. Thermal Stability Evaluation of LEW Gel

Differential scanning calorimetry (DSC) measurements of LEW gel samples were performed using a Phoenix calorimeter (DSC-204-F1, NETZSCH-GerätebauGmbH, Selb, Germany) equipped with an external liquid nitrogen cryostat. Nitrogen was utilized as the purge gas at a flow rate of 20 mL/min. For each measurement, 30 mg of the sample was loaded and sealed into the aluminum pan, and an empty pan was used as a reference sample. The thermogram data were obtained by a heating program ranging from 40 °C to 200 °C at a rate of 8 °C/min.

### 2.12. Texture Profile Analysis (TPA) of LEW Gel

The texture properties of LEW gel were determined by a texture analyzer (TAXT-Plus, Stable Micro System, Godalming, UK), fitted with a flat plunger (model number: SMS-P/6R). The prepared gel samples were compressed twice to 50% of their original height at a crosshead speed of 1 mm/s with a trigger point load of 1 g. Two compression cycles were dwelled between for 5 s. Hardness, adhesiveness, springiness, cohesiveness, gumminess and chewiness were calculated from force–time deformation curves using Texture Expert software version 1.22 (Stable Micro Systems).

### 2.13. Surface Morphology Analysis of LEW Gel

The freeze-dried LEW gel samples were cut into a 1 cm × 1 cm × 1 cm cube and placed on the plate. Samples were then sputter-coated with gold powder in vacuum, and the surface micromorphologies of gels were observed by a scanning electron microscope (SEM) (TM3030, Hitachi high-technologies Corporation, Tokyo, Japan) under an electron accelerating voltage of 15 kV. The imaged photos were produced at 100× magnification.

### 2.14. Swelling Potency Analysis of LEW Gel

To evaluate the swelling properties, approximately 2 g LEW gel samples were placed in enzyme-free simulated gastric juice, which consists of 0.2% sodium chloride and 0.7% hydrochloric acid (37%). The final pH value was 1.2, and the sample was soaked at 37 °C for 150 min. The swollen sample was taken from the solution in intervals of 30 min with the removal of the surface moisture and accurately re-weighed. The swelling ratio was calculated according to the formula as follows:

Swelling ratio (%) = 100 × (W_t_ − W_0_)/W_0_

where W_t_ and W_0_ refer to the weight of the swollen LEW gel at each measuring interval and the accurate weight of the initial LEW gel.

### 2.15. Statistical Analysis

In the present study, three independent trials were carried out for each treatment, and the results were expressed as the mean ± standard deviation (S.D.). A one-way analysis of variance (ANOVA), followed by Duncan’s multiple range test, was used to determine the significant difference between samples at the *p* < 0.05 level.

## 3. Results and Discussion

### 3.1. Surface Hydrophobicity and Free Sulfhydryl Content

**Figure 1:**
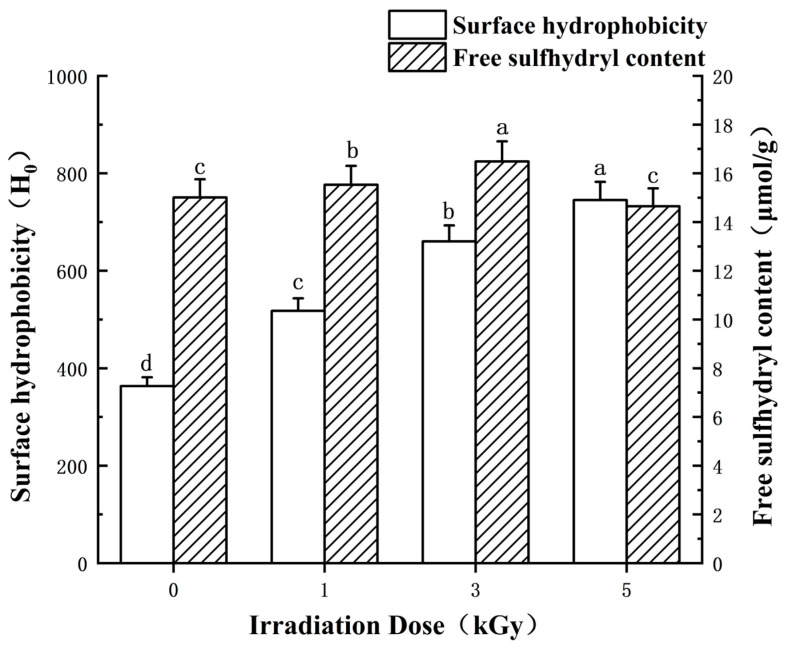
Effects of different irradiation doses (0–5 kGy) on protein surface hydrophobicity (expressed as H_0_) and changes in free sulfhydryl content of LEW samples. For each parameter, means denoted with different lowercase letters differed significantly (*p* < 0.05).

The alteration in free sulfhydryl (SH group) content and the surface hydrophobicity of LEW following irradiation treatment is illustrated in Figure 1. The free SH groups of LEWPs gradually increased and reached a maximum value (~16 μmol/g) at a dose of 3 kGy. As the irradiation dose increased, a significant rise in the surface hydrophobicity of the LEW samples was observed (*p* < 0.05). The irradiation process enhanced the hydrophobicity of proteins by exposing non-polar groups, such as an increase in sulfhydryl content, thereby favoring protein proximity and aggregation tendency [24]. In a similar study, Liu et al. also noted a slight elevation in the surface hydrophobicity of ovalbumin compared to natural ovalbumin across varying irradiation doses [25]. It was also noteworthy that the content of free SH groups of LEW decreased at 5 kGy irradiation (*p* < 0.05) compared with the sample irradiated at 3 kGy but was identical to nonirradiated LEW (*p* > 0.05). This phenomenon implied the excessive loss of active SH during the irradiation-induced oxidation process [5,26]. Overall, these hydrophobic amino acid residues (e.g., Trp, Tyr, Met, Cys, Phe and Pro) tend to be exposed to a more polar environment following irradiation and may facilitate the spontaneous adsorption process of proteins. Indeed, an appropriately increased surface hydrophobicity could show an advantageous factor towards the desirable foaming capacity of proteins [27].

### 3.2. Viscosity

**Figure 2:**
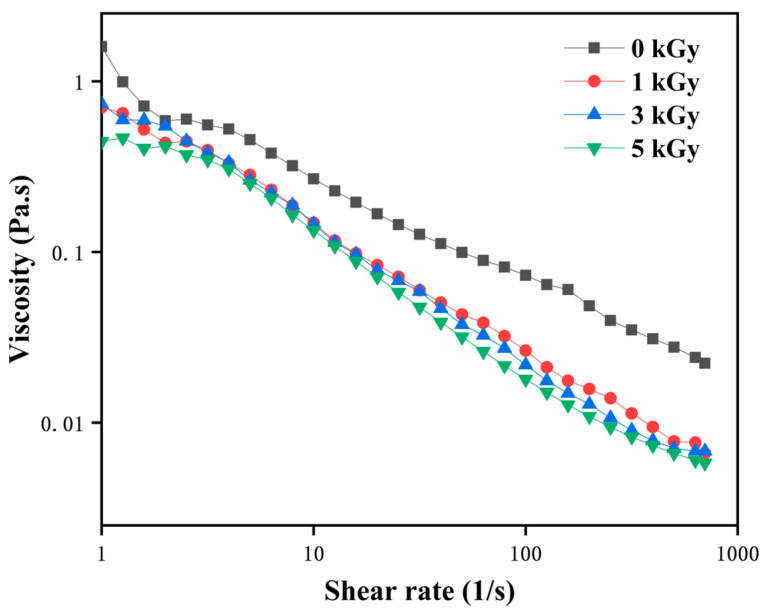
Effects of different irradiation doses (0–5 kGy) on viscosity of LEW.

LEW is characterized as a pseudoplastic fluid, exhibiting a decrease in viscosity as the shear rate increases [28]. The findings presented in Figure 2 demonstrated that the pseudoplastic nature of LEW remained almost constant following irradiation, with a significant decrease in viscosity observed in irradiated samples (*p* < 0.05). Min et al. reported that all egg white became watery even for shell eggs irradiated at 1.0 kGy, but the viscosity changes in egg white were independent of irradiation dose [29]. It is postulated that this reduction in viscosity may be attributed to two potential factors: (1) irradiation could induce a decrease in LEWP viscosity through peptide chain breakage, and (2) irradiation treatment may cause molecular crowding or interactions among LEWPs, leading to the formation of extensive protein networks and subsequently resulting in heightened solution viscosity [30]. Ovomucin, one of the major proteins in egg white, plays an important role in the gel-like structure of egg white. The protein matrix of LEW subjected to irradiation is likely to induce the formation of an ovomucin complex, resulting in a loss of the gel-like nature [31]. However, Song et al. found that varying irradiation doses (1, 2, 5 kGy) did not alter the viscosity of egg white powder [32], indicating that water content plays a crucial role in influencing LEWP viscosity.

### 3.3. Interfacial Tension

**Figure 3:**
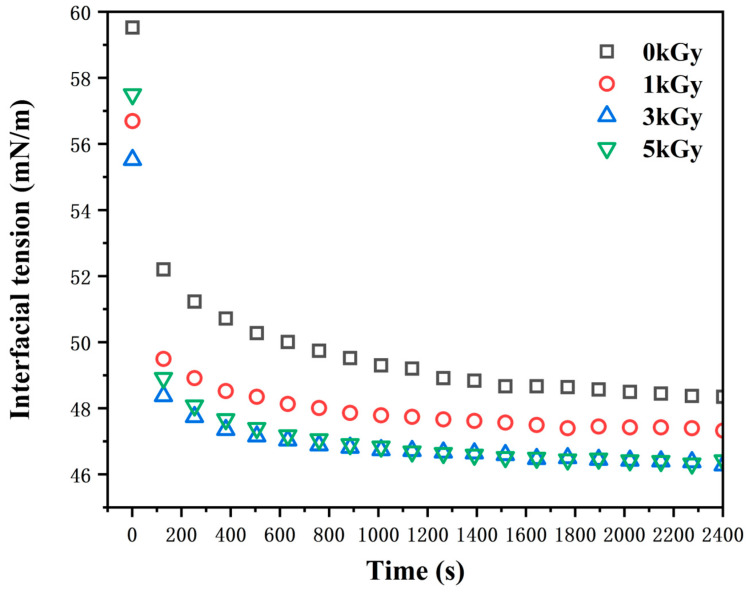
Effects of different irradiation doses (0–5 kGy) on interfacial tension (expressed as mN/m) of LEW samples.

To show more potency in the impact of irradiation on protein viscosity, the interfacial tension of the LEW samples was assessed. It was suggested that the reduction in the viscosity of irradiated egg white could lead to a decrease in surface tension [32]. As depicted in Figure 3, irradiation at low doses (≤3 kGy) notably reduced the interfacial/surface tension of LEW (*p* < 0.05), though the values shared similarity between the samples irradiated at 3–5 kGy irradiation. We hypothesized that the augmentation in surface hydrophobicity, attributed to the adsorption and reorganization of proteins at the interface, contributed to the observed decrease in the surface tension of LEW. Once the adsorption force reaches saturation, the interfacial tension stabilizes. The reduction in surface tension usually results in an increased liquid surface area, thereby enhancing the foaming capacity [33].

### 3.4. Microrheological Behavior

**Figure 4:**
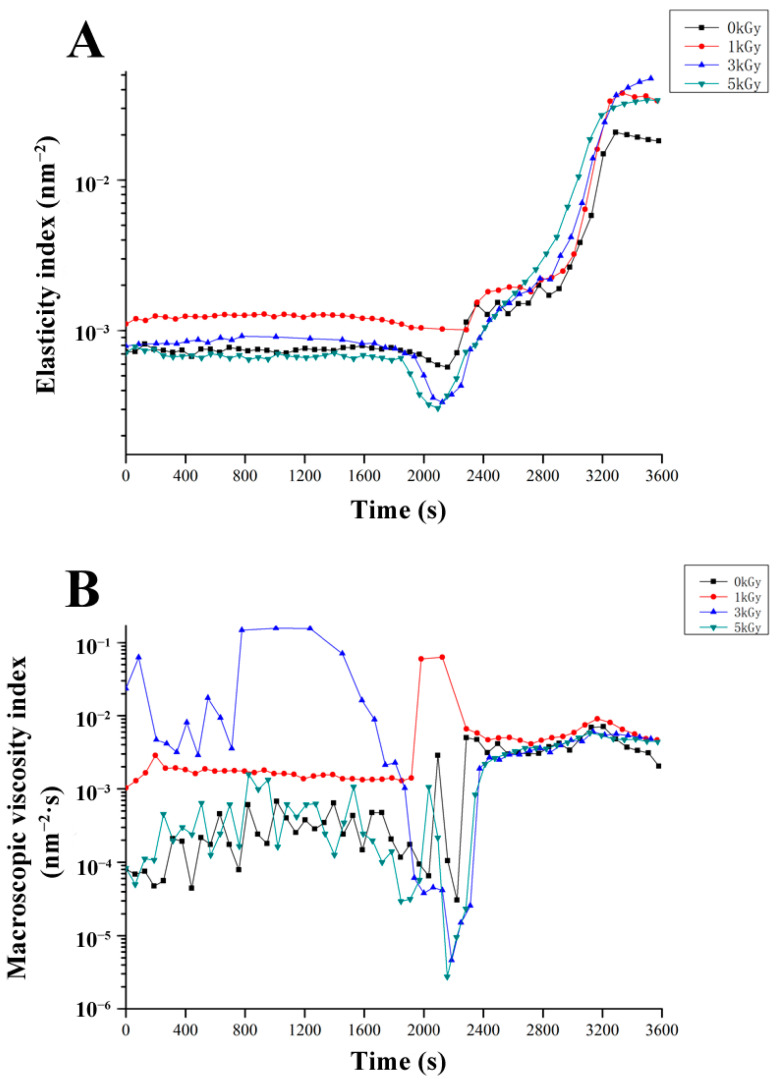
Dynamic changes in EI index (**A**) and MVI value (**B**) of LEW heat-set gels as affected by irradiation doses (0–5 kGy).

The technique of DWS is also referred to as microrheology which offers advantages over traditional mechanical rheology by eliminating the need for mechanical shear force application on the sample [34]. The gel strength, characterized by the relatively stable EI value, corresponds to the inverse of the height of the MSD platform, which is the inverse of the distance required for a droplet to touch the “cage mesh”. The analysis depicted in Figure 4A reveals that the fabricated LEW gel treated with 1 kGy irradiation displayed an apparently higher EI compared to other groups (*p* < 0.05) during the initial phase of heating, primarily attributed to repulsion between protein particles, indicating superior elasticity. As the heating temperature continuously increased, the EI of all samples exhibited a significant rise (*p* < 0.05), and the EI of irradiated LEW samples remained higher than that of the control group. However, unlike the heat-set trail, the EI value of LEW emulsion monitored at room temperature was significantly compromised following ≥3 kGy irradiation compared with the nonirradiated group [18]. We assumed that the fragmentation effect induced by the high irradiation dose and the thermal-induced reorganization pathway of dissociated LEWP components on the viscoelastic interface of oil/water phases might play a role in the formation of the gel network.

Another calculated parameter MVI serves as a quantitative measure of the sample’s macroscopic viscosity. As shown in Figure 4B, low-dose irradiation (1, 3 kGy) during the early stage of gel formation (<2400 s of monitoring) exhibited evident macroscopic viscosity. This result suggests the fluidity of the protein droplets was weakened due to the higher viscosity of the continuous phase for LEW samples irradiated at moderate doses (≤3 kGy). This phenomenon can result from the collision of smaller particles during the heating process, leading to their re-aggregation into larger particles [35]. Lysozyme and other negatively charged proteins from LEW can form large aggregates due to electrostatic interaction which is crucial for the thick viscoelastic interface film and consequently foaming stability [36,37]. In addition, from the combined results of surface hydrophobicity (Figure 1), the enhanced hydrophobic interaction between LEWP molecules may also account for the substantial increase in the macroscopic viscosity of irradiated LEW samples. Nevertheless, up to 5 kGy irradiation benefited minorly for the macroscopic viscosity of LEWPs perhaps because of the destruction of inter-molecular interactions for protein fragments.

### 3.5. Crystalline and Protein Molecular Conformation

The XRD pattern clearly indicated the amorphous region at 2θ = 20° which corresponded to the absorption peaks of ovalbumin (Supplementary Figure S1). Irradiation doses increased, and the peak at 30° became more pronounced, aligning with findings from Xue et al. [38]. These results indicated that irradiation affected the conformation and crystalline structures of LEWPs, implying the variation in the aggregation and crosslinks of different degrees [39].

**Figure 5:**
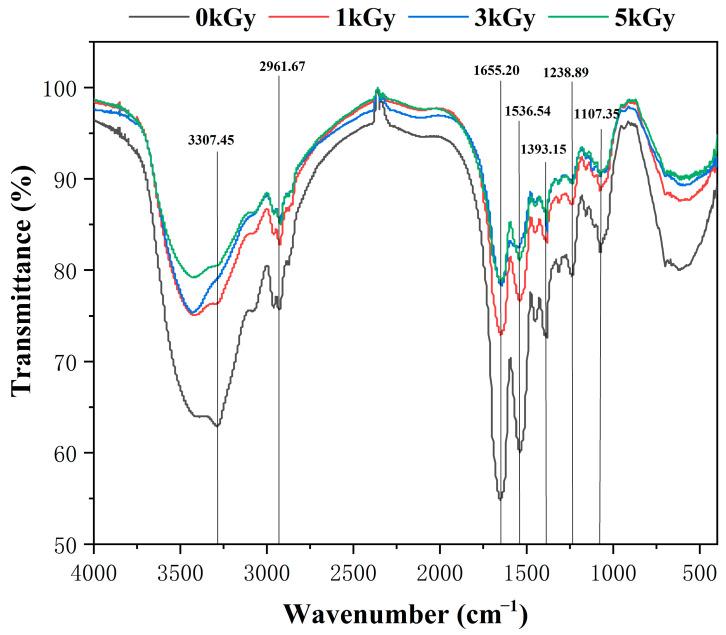
FT-IR pattern of LEW heat-set gels following different doses of irradiation (0–5 kGy).

Xue et al. conducted a study that delved into the crystal structure of the LEW gel, where FT-IR spectroscopy was used to assess the compositional and conformational properties associated with the gel network [40]. In the literature, the peak observed at 2961 cm^−1^ corresponds to the stretching vibration of the CH_3_ and CH_2_ functional groups. Noteworthily, the absorption peaks of the protein encompass the amide I band (1600–1750 cm^−1^), amide II band (1575–1480 cm^−1^) and amide III (1400–1200 cm^−1^). Amide I vibration is contingent upon the secondary structure of the main chain. As depicted in Figure 5, the increase in the radiation dose induced significant alterations in the peak within the amide I region band (1655.2 cm^−1^), which indicated substantial changes in the secondary structure. Additionally, the peak at 1536.5 cm^−1^ (-CN vibration) tended to be compromised with increasing irradiation doses. Furthermore, the absorption peak of the nonirradiated sample (0 kGy) within the 3800–3100 cm^−1^ range, which corresponded to the stretching of the bound hydroxyl, is notably broader compared to that of LEW gel samples following irradiation. Generally, up to 5 kGy irradiation offered no significant alterations in the chemical composition of LEW gel samples.

### 3.6. DSC Pattern

**Figure 6:**
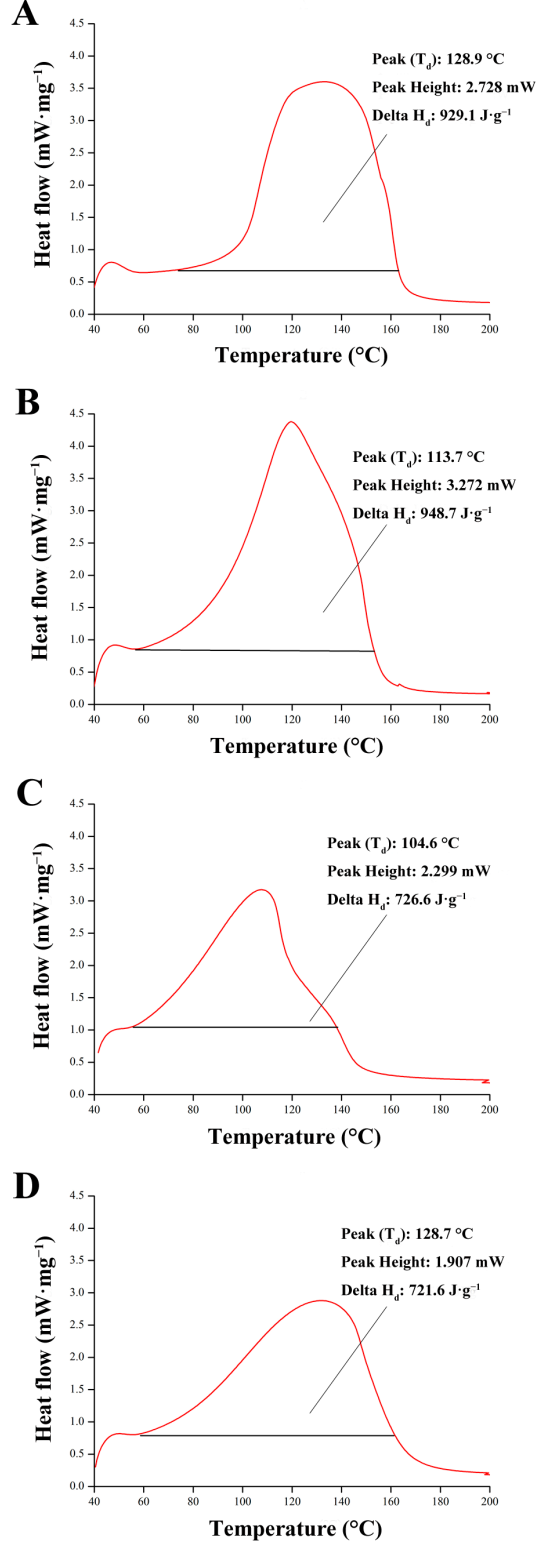
Comparative DSC spectra of LEW heat-set gels following different doses of irradiation ((**A**) 0 kGy, (**B**) 1 kGy, (**C**) 3 kGy and (**D**) 5 kGy).

Figure 6 illustrates the alterations observed in the DSC patterning of LEW gels following different doses of irradiation. It can be seen that a distinct endothermic peak was identified in Figure 6C, indicative of protein denaturation occurring in LEW samples. The characteristic endothermic (T_d_) peaks for the LEW gel after treatment at 0 kGy, 1 kGy, 3 kGy and 5 kGy were recorded at 128.9 °C, 113.7 °C, 104.6 °C and 128.7 °C, respectively, showing a decrease in temperature with escalating irradiation doses. Consequently, the enthalpy (Delta H_d_) associated with these endothermic peaks exhibited a reduction in magnitude as the radiation dose increased (Figure 6). In line with previous findings [30], it can be inferred that at an irradiation dose of 5 kGy, the LEW protein tends to depolymerize, leading to an enhanced fragmentation effect and a decrease in enthalpy change. Li et al. observed that the filamentous aggregate structure of porcine myofibril was entirely disrupted at >5 kGy irradiation, resulting in the disappearance of the peak and the emergence of new peaks associated with myosin and putative actin complexes [41]. Similarly, in the present study, this progression may be interpreted as the initial formation of protein complexes induced by 3 kGy treatment, followed by substantial depolymerization at higher doses (5 kGy).

### 3.7. Foaming Power and Stability

**Figure 7:**
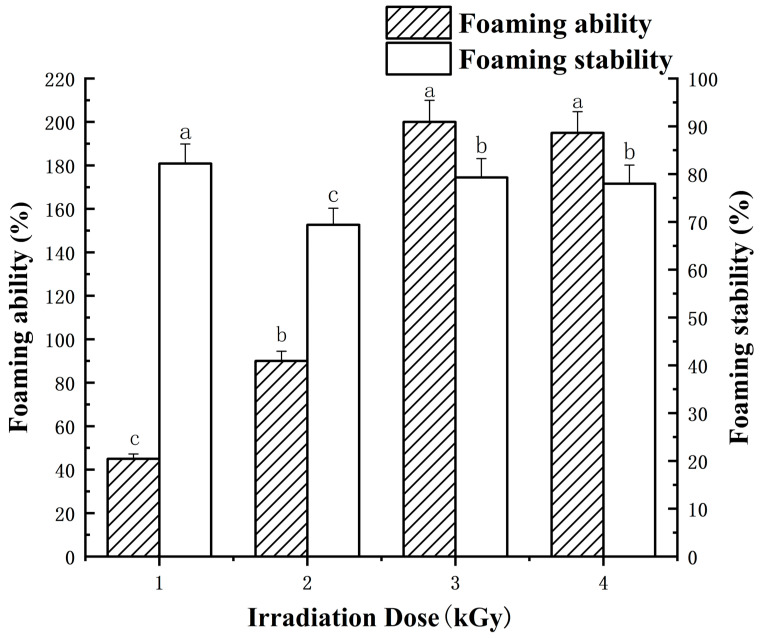
Effects of different irradiation doses (0–5 kGy) on foaming ability/stability of LEWPs. For each parameter, means denoted with different lowercase letters differed significantly (*p* < 0.05).

The impact of irradiation on the foaming capacity and stability of LEW is illustrated in Figure 7. It was evident that irradiation had a notable influence on the foaming properties of LEW. After irradiation, there was a marked increase in the foaming power of LEW as the irradiation dose escalated from 0 kGy to 3 kGy (Figure 7). This result aligns with previous research by Liu et al. [31], who demonstrated a significant enhancement in the foaming power and foam stability of isolated LEW for fresh chicken eggs irradiated at 1.0 and 2.0 kGy, respectively, when compared to the nonirradiated sample.

The enhancement in the foaming capacity of the protein is primarily attributed to the reorganization of β-sheet structures and the enrichment of free/active sulfhydryl [42]. Additionally, the rise in viscosity played a crucial role in bolstering the stability of the foam. Consequently, it is postulated that irradiation may interact with water molecules, leading to the generation of free radicals in the aqueous medium. These radicals could then react with amino acids, inducing protein denaturation [41]. Denatured LEWPs with more flexible conformation show a higher propensity to adhere to the bubble surface compared to natural ones, thereby augmenting protein foaming ability and foam stability [24].

### 3.8. Textural Properties and Microstructure of LEW Heat-Set Gels

The texture nature of LEW heat-set gel products serves as a crucial sensory attribute for food consumers. The analysis depicted in Supplementary Table S1 revealed that the hardness (firmness) of LEW gels ranged from 255.2 ± 12.6 to 269.6 ± 10.7 gf without any significant differences between them (*p* > 0.05), whereas irradiation induced a gradual increase in gel springiness (elasticity) with a maximum value of 3.8 ± 0.1 at 5 kGy (*p* < 0.05). Specifically, exposure to 1 kGy irradiation led to a significant reduction in the adhesion of the LEW gel (*p* < 0.05), reaching a minimum value of −59.1 ± 3.0 (Supplementary Table S1). Similar evidence has been found in shell eggs irradiated at low doses (≤2.5 kGy) regarding the marginal effect on the gel firmness of egg white and the thermal characteristics of egg white products [10,29,32]. It should be noted that the collected data of the gel texture profile may be greatly affected by the water state and activity of LEWPs as well as other possible technical factors such as the aerobic and temperature condition of protein samples during the irradiation process [5,29,32,43]. Together, irradiation has the potential to enhance heat-induced gelation and facilitate the development of a structurally sound matrix.

**Figure 8:**
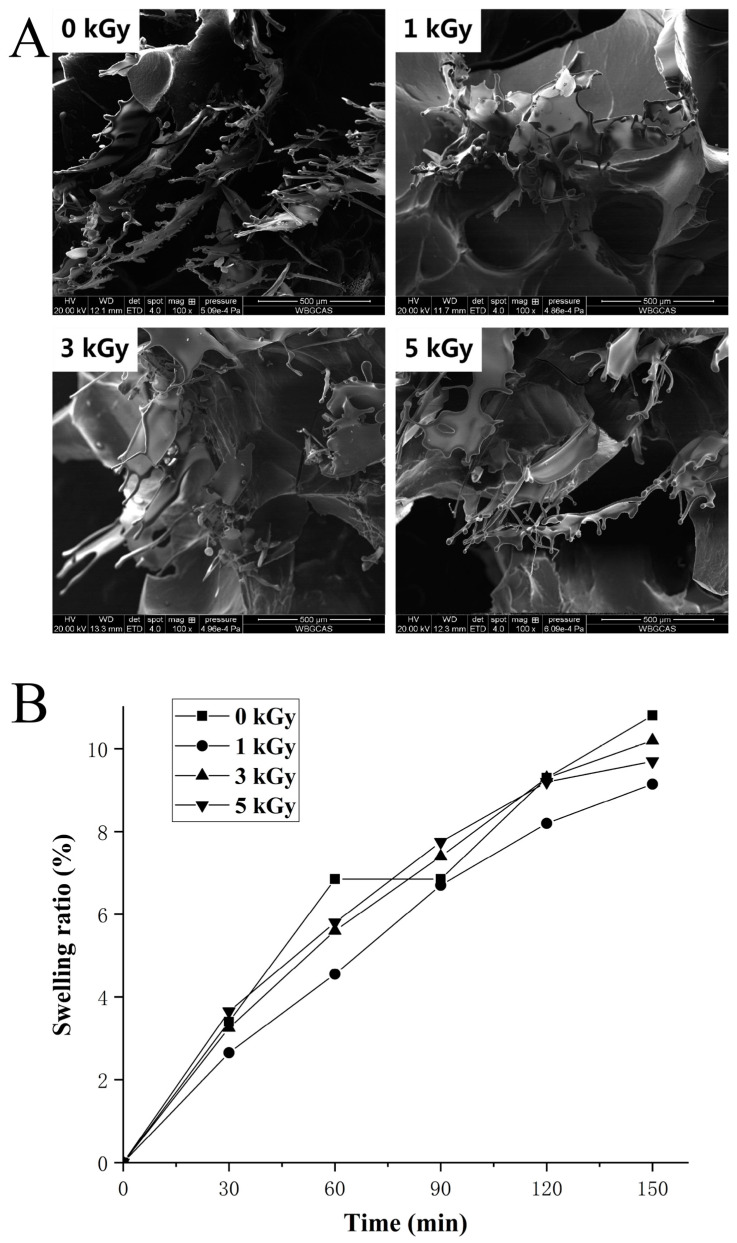
(**A**) SEM images (100× magnification) of LEW heat-set gels following different doses of irradiation (0–5 kGy). (**B**) Effect of different irradiation doses (0–5 kGy) on swelling degree of LEW heat-set gels subjected to enzyme-free simulated gastric juice.

We further examined the network microstructure of gels following irradiation treatment through SEM methodology. The analysis revealed a three-dimensional network structure in all samples, characterized by a loosely arranged linear fiber structure (Figure 8A). Upon irradiation at 1 kGy, a significant increase in aggregates within the amorphous region was observed compared to nonirradiated samples (Figure 8A). Interestingly, the 5 kGy irradiated sample exhibited smaller crystals than the LEW gel sample subsequent to 3 kGy irradiation, potentially due to the enhanced gel elasticity as depicted in Supplementary Table S1. The irradiation-induced decomposition of water led to the generation of hydroxyl groups, facilitating protein modification and entanglement. Previous research has also indicated that oxidized animal proteins can aggregate and form fiber structures within the gel matrix enriched by non-covalent interactions (e.g., hydrophobic interaction) [31,41,44].

### 3.9. Swelling Potency of LEW Heat-Set Gels

Figure 8B illustrates the swelling phenomenon observed in LEW heat-set gel when exposed to enzyme-free simulated gastric juice. The capacity of proteins to absorb water is a critical factor, particularly in applications of nutritional drug delivery, as it can regulate the release rate of active components in gastrointestinal environments. Towards protein gel swelling, the disparity in osmotic pressure between the gel and the surrounding aqueous medium can be noted [45,46,47]. The data presented in Figure 8B indicate a notable lowest swelling capacity of the LEW gel sample following 1 kGy irradiation amongst all assayed groups. In particular, within a 150 min timeframe, the swelling of the LEW gels following ≥3 kGy irradiation had a comparable potency compared to the nonirradiated control (Figure 8B). This result was not surprising as an evident macroscopic viscoelastic interface of LEWPs irradiated at a low dose (<3 kGy) can be formed at the early stage of gelation (Figure 4). The swelling of protein hydrogels depends on the microstructure, stranded or particulate. In egg white stranded gels, covalent disulfide bonds appear to be critical during swelling where the crosslinking density of the protein scaffold readily influences the acidification availability [45]. Prior to the gelation of LEW, the result of free SH groups supported the steady modification of the protein surface by moderate irradiation treatment (≤3 kGy) followed by the excessive oxidation of SH groups occurring at 5 kGy irradiation probably due to conversion into disulfide (S-S) bonds (Figure 1). Together with the SEM result (Figure 8A), it was reasonable to suggest that 1 kGy irradiation gave rise to the new protein aggregates mainly with physical entanglements during structural collapse, which would hinder the extensive swelling of the gel network. Nevertheless, the chemical interactions (e.g., the S-S bonds) between LEWPs irradiated at high doses (≥3 kGy) may play a role in the maintaining the elastic mechanical properties of swollen hydrogels during the follow-up acidification at pH 1.2 (Figure 8B).

## 4. Conclusions

This research systematically investigated the impact of varying doses of γ-ray irradiation on the foaming and heat-set gel characteristics of LEW. Through the examination of parameters such as viscosity, surface hydrophobicity, surface tension, scanning electron microscopy and microrheology, it was observed that irradiation led to notable enhancements in foaming and heat-induced gel properties. The evidence of the physicochemical and microstructural properties of LEW suggested the co-occurrence of protein unfolding/fragmentation and aggregation (mainly as a non-covalent interaction) during low doses (≤3 kGy) of irradiation. Specifically, the structural collapse state at ≤3 kGy irradiation might be the key factor significantly improving the foaming capacity of LEW. Meanwhile, a lower viscosity of LEW caused by ≤3 kGy irradiation should be desirable for the industrial application of the effective separation of egg white and yolk during the egg breaking process. Additionally, high doses of irradiation (≥3 kGy) induced smaller protein crystal structures of LEWPs in terms of the heat-set gel nature but had no compromise on the swelling potency. These findings provide good suggestions for the dosage selection of γ-ray irradiation in enhancing the functional properties of egg white products, though further comprehensive investigations in the line of protein digestibility and health benefits are still warranted.

## Data Availability

The original contributions presented in the study are included in the article/Supplementary Materials, further inquiries can be directed to the corresponding authors.

## Supplementary Materials

The following supporting information can be downloaded at: https://www.mdpi.com/article/10.3390/foods13091342/s1, Figure S1: XRD spectra of LEW heat-set gels following different doses of irradiation (0–5 kGy); Table S1: Effects of different irradiation doses (0–5 kGy) on the textural properties of LEW heat-set gels.
