# Supplementary material for: Irradiation-Assisted Enhancement of Foaming and Thermal Gelation Functionality of Liquid Egg White
Source: Foods. 2024 Apr 26;13(9):1342. doi: 10.3390/foods13091342 (PMC11083238; doi:10.3390/foods13091342)
Supplement: Supplementary file 1 [file foods-13-01342-s001.zip › foods-2954592-supplementary.pdf]

*Supporting Information*

# Irradiation-assisted enhancement of foaming and thermal gelation functionality of liquid egg white

**Yan Zhang [1], Jianying Zhao [2], Lichao He [1], Jin Zhu [1], Yue Zhu [1], Guofeng Jin [1,*], Ruihang Cai [3,*], Xiaola Li [3], Chengliang Li [1,4]**

[1] Key Laboratory of Geriatric Nutrition and Health (Ministry of Education), China Food Flavor and Nutrition Health Innovation Center, School of Food and Health, Beijing Technology and Business University, 100048 Beijing, China

[2] Department of Tea and Food Science and Technology, Jiangsu Vocational College of Agriculture and Forestry, Jurong 212400, China

[3] Zhejiang Institute of Subtropical Crops, Zhejiang Academy of Agricultural Sciences, Wenzhou, 325005, China

[4] College of Food Science & Technology, Huazhong Agricultural University, Wuhan, 430070, China

* Co-corresponding authors.: jinguofeng@btbu.edu.cn (Dr. Guofeng Jin); cairh@zaas.ac.cn (Dr. Ruihang Cai).

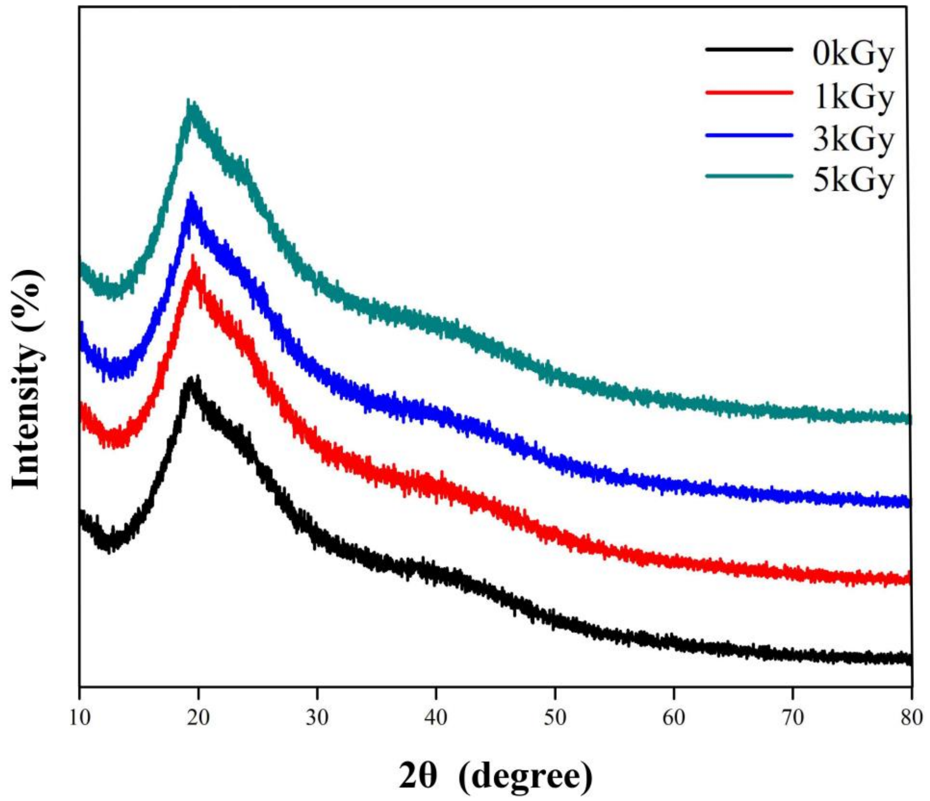


**Figure S1.** XRD spectra of LEW heat-set gels following different doses of irradiation (0-5 kGy).

**Table S1**. Effects of different irradiation doses (0-5 kGy) on the textural properties of LEW heat-set gels.

| **Irradiation dose (kGy)** | **Hardness (gf)** | **Adhesiveness (-)** | **Springiness (-)** | **Cohesiveness (-)** | **Gumminess (-)** | **Chewiness (-)** |
|---|---|---|---|---|---|---|
| 0 | 261.3 ± 7.2$^{a}$ | 146.4 ± 3.1$^{a}$ | 1.0 ± 0.0$^{c}$ | 0.6 ± 0.0$^{a}$ | 145.5 ± 1.6$^{a}$ | 137.4 ± 1.4$^{d}$ |
| 1 | 269.6 ± 10.7$^{a}$ | -59.1 ± 3.0$^{c}$ | 3.5 ± 0.1$^{b}$ | 0.5 ± 0.2$^{b}$ | 132.4 ± 3.6$^{b}$ | 461.2 ± 5.2$^{b}$ |
| 3 | 255.2 ± 12.6$^{a}$ | -36.4 ± 3.5$^{b}$ | 3.7 ± 0.1$^{a}$ | 0.5 ± 0.3$^{b}$ | 119.8 ± 2.6$^{c}$ | 445.3 ± 5.3$^{c}$ |
| 5 | 260.6 ± 3.2$^{a}$ | -38.9 ± 5.7$^{b}$ | 3.8 ± 0.1$^{a}$ | 0.5 ± 0.2$^{b}$ | 129.3 ± 4.9$^{b}$ | 484.1 ± 14.2$^{a}$ |

Note: Means in the same column (corresponding to the same parameter) not followed by a common superscript letter differ significantly ($P < 0.05$).
